# Study on Spatial and Temporal Distribution Characteristics of Coordinated Development Degree among Regional Water Resources, Social Economy, and Ecological Environment Systems

**DOI:** 10.3390/ijerph16214213

**Published:** 2019-10-30

**Authors:** Xinkui Wang, Zengchuan Dong, Wei Xu, Yun Luo, Tao Zhou, Wenzhuo Wang

**Affiliations:** College of Hydrology and Water Resource, Hohai University, Nanjing 210098, China; xuwill@hhu.edu.cn (W.X.); luoyun_hhu@hhu.edu.cn (Y.L.); 160401010016@hhu.edu.cn (T.Z.); wzwang25@hhu.edu.cn (W.W.)

**Keywords:** coordinated development degree, restriction mechanism, evaluation index system, spatial and temporal changes

## Abstract

Water resources utilization, social economy development, and ecological environment protection are key factors in regional sustainable development. Scientific evaluation of regional coordinated development status and diagnosis of regional uncoordinated development constraints will improve the management level of decision-makers. At present, most developing countries have the problem of unbalanced regional development caused by the one-sided pursuit of a certain system. Taking 14 prefecture-level cities in Hunan Province as cases, this paper analyzed the spatial and temporal distribution characteristics of the carrying capacity level of the water resources system, the development level of the social economy system and the protection level of the ecological environment system in each evaluation unit based on entropy weight method and order parameter analysis. Based on the theory of coordinated development, a calculation model of a coordinated development degree was constructed, and the corresponding evaluation criteria were formulated. The spatial and temporal distribution characteristics of a coordinated development degree in each research unit were analyzed and evaluated. The results showed that the average coordinated development degree of Hunan Province from 2004 to 2016 evolved from “Light disorder recession” to “Nearly disorder recession”, then to “Reluctance coordinated development”. Restricted by different systems, the coordinated development degree in each research unit presented spatial and temporal differences. According to different development stages and the characteristics of different regions, corresponding development strategies can be formulated to provide the guidance for coordinated the development of regions.

## 1. Introduction

In recent years, with rapid economic development and population expansion, human beings are facing challenges from ecological pollution and resource depletion [1,2,3]. The rapid development of social economy has raised a series of problems, such as water shortages, water pollution, wetland destruction, and habitat fragmentation [4,5,6,7,8]. Therefore, it is of great strategic significance for regional sustainable development to study the coordinated development of social economy development, natural resource utilization, and ecological environment protection [9,10]. Regional coordinated development is a complex issue involving multiple elements, including society, economy, resources, ecology, and environment. These elements work and cooperate with each other to achieve the optimal allocation of regional resources, the maximization of economic, social, ecological and environmental benefits [11,12].

As an important natural resource, water resources are widely used in human production and living activities. Water resources are not only basic natural resources, but also strategic economic resources and ecological control factors [13,14,15]. Previously, plenty of studies focused on the coordinated development of the water resources system and the social economy system. Jiang et al. [16] calculated the coupling degree and coupling coordination degree of water resource carrying capacity of Shenyang City using the capacity coupling model, in which the pressure of social and economic development on water resource system and the supporting capacity of water resource system on social and economic development were compared and analyzed. Zhou et al. [17] improved the traditional coupling coordination model by combining the full displacement polygon comprehensive mapping method (FPPSI) with the finite element method. They investigated the coordinated development relationship between the social-economic system and the water resources system in the Taihu Lake basin. Du et al. [18] calculated the comprehensive development level of the two systems in Hexi Corridor based on the synergy theory. The fuzzy evaluation method was used to evaluate the coordinated development of the two systems.

In previous studies, most research mainly focused on the coordinated development between the water resources system and the social-economic system [19,20,21]. However, in some cases, a coordinated development status of two systems can only be achieved at the expense of compromise and even decline in other systems. It is well-known that ecosystem degradation is a major cause of the ongoing challenges to regional development. Since 1900, about 64% to 71% of the world′s natural wetlands have disappeared due to human activities [22]. The deterioration of the ecological environment has caused the demise of many ancient civilizations in history. Therefore, it is necessary to integrate the ecological environment system into the process of regional coordinated development while coordinating the stable development of social economy and efficient utilization of water resources. We need to diagnose the development state of the water resources system, the social economy system, and the ecological environment system in different regions and in different periods of time, and their coordinated development state. In addition, we need to find out which system is lagging behind in its development and thus affects the coordinated development of the region. By doing so, we can provide decision-makers with effective suggestions to promote regional coordinated development.

Therefore, in this paper, a novel calculation model of coordinated development degree combined with water resources system, social economy system, and ecological environment system was developed. In our study, Hunan Province was selected due to the prominent contradiction among the utilization of water resources, the development of social economy, and protection of the ecological environment. There are four aims in this study: (1) To solve the problem of one-sided pursuit of the development of one or two systems while ignoring the overall coordinated development of the region, (2) to reveal the spatial and temporal distribution characteristics of water resource system, social economy system, ecological environment system and the coordinated development degree of the three systems in Hunan Province, (3) to diagnose the restriction relationship of uncoordinated development in different regions and development stages of Hunan Province, (4) to formulate corresponding development strategies according to different development stages and characteristics of different regions to provide reference for coordinated development of region.

## 2. Materials and Methods

### 2.1. Overview of Research Area and Data Sources

Hunan Province (east longitude 108°47′~114°15′, north latitude 24°38′~ 30°08′), is located in the middle of China. Its territory is surrounded by mountains on three sides, with a total area of 21.18 km^2^. In the north part, it faces the opening of a horseshoe landform. Terrain types include plains, basins, hills, and mountains. Hunan Province crosses the Yangtze River and the Pearl River drainage. Hunan Province belongs to subtropical monsoon climate, with an average annual rainfall of 1450 mm, an average annual water resource of 168.9 billion m³, an average annual surface water resource of 168.2 billion m³, and an average annual groundwater resource of 39.15 billion m³ (non-repeating volume was 700 million m³). 

As one of the important provinces in the strategic development of “the rise of the central region” in China [23,24], Hunan Province shoulders the important task of maintaining the sustainable development of social economy. With the total water resources of Hunan Province ranks the sixth in China, and the per capita water resource of 2500 m³ (slightly higher than the national level), the conditions for the development and utilization of water resources are good. However, in recent years, with the increase of water consumption in the process of urbanization and industrial and agricultural irrigation, wastewaters are directly discharged into rivers. The COD, ammonia nitrogen content and other indicators in the rivers are exceeding the standards, leading to an increase in ecological and environmental problems and the pressure on sustainable development [25].

To facilitate the data collection, calculation, analysis and policy making in each administrative region, 14 prefecture-level administrative regions in Hunan Province were taken as research units, including Changsha, Zhuzhou, Xiangtan, Hengyang, Shaoyang, Yueyang, Changde, Zhangjiajie, Yiyang, Chenzhou, Yongzhou, Huaihua, Loudi, and Xiangxi.

In this study, the original data of the water resources system were derived from Hunan Province hydrological statistical yearbook [26]. The original data of the social economy system were derived from Hunan Province statistical yearbook on national economic and social development [27]. The original data of the ecological environment system were derived from Hunan Province statistical yearbook on forestry [28] and Hunan Province statistical yearbook on water resources [26]. Some of the indicators were converted from the related calculation formula and initial data. Per capita water consumption was calculated by dividing the total water consumption by the total population. Per capita water resources were calculated by dividing the total water resources by the total population.

### 2.2. Comprehensive Development Evaluation of Three Systems

#### 2.2.1. Selection of Comprehensive Development Evaluation Indexes

The basis for analyzing the coordinated development degree of the three systems is to construct a comprehensive development evaluation index system to quantitatively calculate the carrying capacity level of the water resources system, development level of social the economy system, and protection level of the ecological environment system. This paper follows the principles of scientific, systematic, hierarchical, and operable, and combines the structural characteristics of the research area. Then we selected per capita water consumption, water consumption per 10^4^ CNY of GDP, annual rainfall, per capita water resources and total water resources per unit as the evaluation indexes to reflect the comprehensive carrying capacity level of water resources system. We selected per capita income, urbanization rate, per capita GDP, and population density as the evaluation index to reflect the comprehensive development level of social economy system. Next, we selected wetland protection rate, COD concentration in the channel, forest coverage, and ecological compliance rate of water functional zone as the evaluation indexes to reflect the comprehensive protection level of ecological environment system [29,30,31,32,33]. The comprehensive development evaluation index system of the three systems is shown in Table 1.

#### 2.2.2. Calculation of Comprehensive Development Evaluation Index

In this study, a mature and universal entropy weight method is selected to calculate the index weight, which can make the process clear, quantitative, and qualitative, and requires fewer data. The entropy weight method calculates the information entropy of indicators and determines the weight of indicators according to the influence of the relative change degree of indicators on the whole system. Indicators with a large relative change degree have a large weight. This method is widely used in various fields [34,35,36].

The specific steps are as follows [37,38]:

1. Determine the evaluation object, establish the evaluation index system and construct the index matrix $X_{m\times n}$
  (1) $$X_{m\times n}={\left[\begin{array}{cccc} x_{11} & x_{12} & \ldots & x_{1n}\\ x_{21} & x_{22} & \ldots & x_{2n}\\ \ldots & \ldots & \ldots & \ldots \\ x_{m1} & x_{m2} & \ldots & x_{mn} \end{array}\right]}_{m\times n}$$
  where m stands for the total number of the evaluation projects i, n stands for the total number of the indicators j.
2. Standardize the index matrix $X_{m\times n}$ to get the standardized matrix $Y_{m\times n}$
  For the positive properties index, then:
  (2) $$y_{ij}=\frac{x_{ij}-min\left\{x_{j}\right\}}{max\left\{x_{j}\right\}-min\left\{x_{j}\right\}}$$
  For the negative properties index, then:
  (3) $$y_{ij}=\frac{max\left\{x_{j}\right\}-x_{ij}}{max\left\{x_{j}\right\}-min\left\{x_{j}\right\}}$$
  where $x_{ij}$ is the actual value of the indicator j in evaluation project i, $max\{x_{j}\}$ means the maximum value of indicator j $min\{x_{j}\}$ means the minimum value of indicator j $y_{ij}$ means the standardized value of the actual value $x_{ij}$.
  Then, the standardized matrix is
  (4) $$Y_{m\times n}={\left[\begin{array}{cccc} y_{11}\; & y_{12} & \;\ldots \; & y_{1n}\\ y_{21}\; & y_{22}\; & \ldots \; & y_{2n}\\ \ldots & \ldots & \ldots & \ldots \\ y_{m1}\; & y_{m2}\; & \ldots \; & y_{mn} \end{array}\right]}_{m\times n}$$
3. Calculate the entropy $E_{j}$ of the indicator j.
  According to the definition of information entropy in the information theory, the information entropy $E_{j}$ of the indicator j.
  (5) $$E_{j}=-\frac{1}{lnm}\overset{m}{\underset{i=1}{\sum }}P_{ij}lnP_{ij}$$
  where $P_{ij}$ is the proportion of the standardized value $y_{ij}$ in the sum of the standard values of the indicator j, $P_{ij}=\frac{y_{ij}}{\sum_{i=1}^{m}y_{ij}}$. If $P_{ij}=0$, then $\underset{p_{ij}\to \infty }{lim}p_{ij}\ln p_{ij}=0$.
4. Calculate the entropy weight $w_{j}$ of the indicator j.

According to the calculation formula of information entropy, the information entropy of each indicator is calculated, and the weight of the indicator j is

(6) $$w_{j}=\frac{1-E_{j}}{n-\sum_{j=1}^{n}E_{j}}$$

### 2.3. Evaluation Model of Regional Coordinated Development Degree

#### 2.3.1. Comprehensive Development Evaluation Indexes of the Three Systems

By using the entropy weight method, the annual comprehensive evaluation value of the three systems in each research unit can be calculated, respectively [37]. The values can represent the carrying capacity level of the water resources system, the development level of the social economy system, and the protection level of the ecological environment system. The carrying capacity level of the water resources system (CCLWR), the development level of the social economy system (DLSE) and the protection level of the ecological environment system (PLEE) are calculated by the following formulas:

(7) $$CCLWR\left(t,u\right)=\overset{l}{\underset{i=1}{\sum }}\omega_{WR,i}y_{WR,i,t,u}$$

(8) $$DLSE\left(t,u\right)=\overset{m}{\underset{j=1}{\sum }}\omega_{SE,j}y_{SE,j,t,u}$$

(9) $$PLEE\left(t,u\right)=\overset{n}{\underset{k=1}{\sum }}\omega_{EE,k}y_{EE,k,t,u}$$

where l, $\omega_{WR,i}$ and $y_{WR,i,t,u}$ represent the total number of indicators in water resources system, the weight of the indicator i in water resource system and the standardized value at time t in research unit u, m, $\omega_{SE,j}$ and $y_{SE,j,t,u}$ represent the total number of indicators in social economy system, the weight of the indicator j in social economy system and the standardized value at time t in research unit u, n, $\omega_{EE,k}$ and $y_{EE,k,t,u}$ represent the total number of indicators in ecological environment system, the weight of the indicator k in ecological environment system and the standardized value at time t in research unit u.

#### 2.3.2. Calculation of Coordinated Development Degree (CDD)

CDD is a quantitative index to describe the degree of coordination of various factors or systems within a region, which reflects the trend of the system from disorder to order [39]. In this paper, the conception of CDD is derived from the theory of regional coordinated development, system theory, and the theory of the minimum deviation coefficient. The CDD of water resources system, social economy system, and ecological environment system refers to the coordinated state of the carrying capacity level of water resources system, the development level of the social economy system and the protection level of the ecological environment system at a certain stage of development, respectively. By constructing the coordination among CCLWR, DLSE, and PLEE, the model diagnoses the coordination degree of the development of three systems in each research unit, making it an important basis for regional development planning [17].

The coordination degree $CD\left(t,u\right)$ of water resources system, social economy system and ecological environment system at time t is:

(10) $$CD\left(t,u\right)={\left[\frac{CCLWR\left(t,u\right)\times DLSE\left(t,u\right)\times PLEE\left(t,u\right)}{{\left(\frac{CCLWR\left(t,u\right)+DLSE\left(t,u\right)+PLEE\left(t,u\right)}{3}\right)}^{3}}\right]}^{\mu }$$

where $CCLWR\left(t,u\right)$, $DLSE\left(t,u\right)$ and $PLEE\left(t,u\right)$ represent the values of the CCLWR, DLSE, and PLEE at time t in research unit u, respectively. The μ represents the adjustment coefficient. This study considered the coordinated development of three systems, so we took μ=3 [40,41].

The coordination degree $CD\left(t,u\right)$ of is the value between 0 and 1. When $CD\left(t,u\right)=1$, the degree of coordination is extremely high, and the system tends to coordinate the orderly structure. When $CD\left(t,u\right)=0$, the degree of coordination is minimal, and the system tends to develop in disorder.

Under certain constraints, the CCLWR, DLSE, and PLEE have a certain upper limit threshold value, that is, the corresponding composite index satisfies $CCLWR\left(t,u\right)+DLSE\left(t,u\right)+PLEE\left(t,u\right)=C$, When $CCLWR\left(t,u\right)=DLSE\left(t,u\right)=PLEE\left(t,u\right)$, the value of $CCLWR\left(t,u\right)\times DLSE\left(t,u\right)\times PLEE\left(t,u\right)$ reaches the maximum, which indicates that the development degree of water resource system, social economy system and ecological environment system is the most coordinated.

Therefore, the development degree $DD\left(t,u\right)$ of water resource system, social economy system and ecological environment system at time t in research unit u is [42]:

(11) $$DD\left(t,u\right)=\lambda_{WR}CCLWR\left(t,u\right)+\lambda_{SE}DLSE\left(t,u\right)+\lambda_{EE}PLEE\left(t,u\right)$$

where $\lambda_{WR}$, $\lambda_{SE}$, $\lambda_{EE}$ represent the undetermined development coefficient of water resources system, social economy system and ecological environment system respectively. $\lambda_{WR}>0$, $\lambda_{SE}>0$, $\lambda_{EE}>0$ and $\lambda_{WR}+\lambda_{SE}+\lambda_{EE}=1$. This study according to “Hunan Province open rise strategy development plan [23]” and the related expert consultation result, which highlight that as an important province in central China, the managers of Hunan Province should put the social and economic construction in a relatively higher position, therefore, we determined that $\lambda_{WR}=0.3$, $\lambda_{SE}=0.4$, $\lambda_{EE}=0.3$.

To sum up, the coordinated development degree $CDD\left(t,u\right)$ of the regional water resource system, social economy system and ecological environment system at time t in research unit u is [43]:

(12) $$CDD\left(t,u\right)=\sqrt{CD\left(t,u\right)\times DD\left(t,u\right)}$$

#### 2.3.3. Classification and Standard Division of Coordinated Development Degree

This study focuses on the CDD among three variables of water resources system, social economy system, and ecological environment system, but there are few relevant studies on unified classification standards at present. Therefore, according to the mathematical model of coordinated development, this study classified the CDD by taking 0.01 as the boundary standard to classify each type of coordinated development [10,40,44] and obtained the classification and evaluation criteria of coordinated development of three systems, which is shown in Table 2.

### 2.4. Research Framework

**Figure 1:**
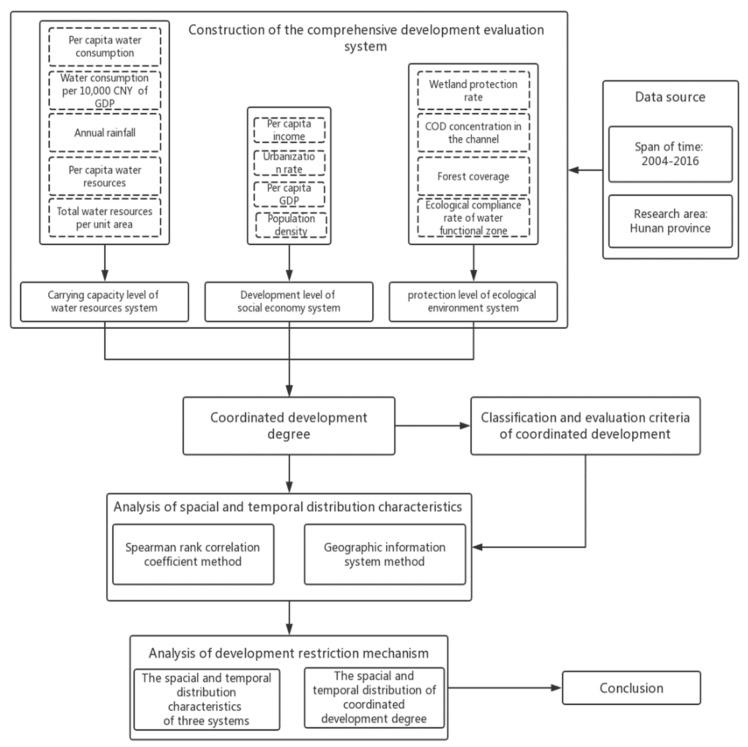
Overview of research framework.

To determine the quantitative relationship and the interaction mechanism between the water resources system, the social economy system and the ecological environment system in Hunan Province, we collected and analyzed data of 14 municipal areas from 2004 to 2016. First, this paper establishes an evaluation system of comprehensive development indexes of the water resources system, social economy system, and the ecological environment system respectively. After comprehensive development evaluation, this paper calculates the coordinated development degree based on the coordinated development theory. Then, we adopt the Spearman rank correlation coefficient method and geographic information system to analyze the spatial and temporal characteristics of the comprehensive development index and the coordinated development degree between the water resources system, the social economy system, and the ecological environment system in Hunan Province. Finally, the paper draws conclusions from the full text and tries to offer scientific suggestions for achieving coordinated and sustainable development of the water resources system, the social economy system, and the ecological environment system. The research framework is shown in Figure 1.

## 3. Results

### 3.1. Weight Calculation Results of Comprehensive Development Evaluation of the Three Systems

According to the basic principle of entropy weight method, entropy is a measurement for the degree of disorder of a system. If the information entropy of the index is smaller, the more information the index provides, and the higher the weight should be [45]. According to formula (1) to (7), the original data of 13 indicators in three systems were taken as input, and the weight value of each indicator can be calculated, as shown in Table 3.

### 3.2. Spatial and Temporal Distribution Characteristics of Comprehensive Development Evaluation of Three Systems

In order to obtain the spatial and temporal distribution characteristics of the CCLWR, DLSE, and PLEE with 13 years in 14 research units, Spearman’s rank correlation coefficient method [46] and geographic information system (GIS) was used to analyze the temporal distribution and the spatial distribution respectively.

Spearman’s rank correlation coefficient method is mainly used to solve the problem of the name data and the sequence data. It is applicable to data with two-column variables and a linear relationship with rank variable nature [47,48].

The specific formula of rank correlation coefficient method is as follows:

(13) $$R_{n}=\left[\frac{6\times \sum_{i=1}^{N}d_{i}^{2}}{N^{3}-N}\right]$$

(14) $$d_{i}=X_{i}-Y_{i}$$

where $R_{n}$ is the rank correlation coefficient, $d_{i}$ is the difference between variable $X_{i}$ and variable $Y_{i}$; $X_{i}X_{i}$ is the sequence number from period 1 to period N in order of evaluation value from small to large, $Y_{i}$ is the sequence number arranged by time, N is the number of samples. In Spearman’s rank correlation coefficient method, the absolute value of the rank correlation coefficient $R_{n}$ is compared with the critical value $W_{p}$ in the rank correlation coefficient statistical table. If $\left|R_{n}\right|\geq W_{p}$, it indicates that the change trend is significant. When $R_{n}$ is positive, it tends to go up, and when $R_{n}$ is negative, it tends to go down. In this study, the time series we selected were from 2004 to 2016, a total of 13 years, and the significance level we selected was 0.01. According to Table 4, the critical value of Spearman’s rank correlation coefficient $W_{p}$ is 0.703.

#### 3.2.1. Spatial and Temporal Distribution Characteristics of the Carrying Capacity Level of the Water Resources System

**Figure 2:**
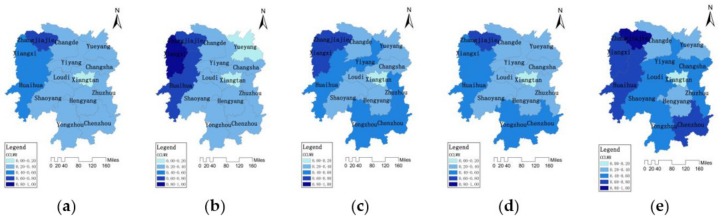
Temporal distribution of CCLWR among 14 cities in Hunan Province during (**a**) 2004, (**b**) 2007, (**c**) 2010, (**d**) 2013, (**e**) 2016.

According to formula (7), the standardized indicator values and entropy weights in the water resource system can be used as the input data to calculate CCLWR. CCLWR takes several indicators into account, such as per capita water consumption, water consumption per 10^4^ CNY of GDP, annual rainfall, per capita water resources, and total water resources per unit, to measure the comprehensive evaluation value of the carrying capacity status of the regional water resources system. The larger the value of the comprehensive evaluation is, the higher the carrying capacity level of the water resources system. The spatial and temporal distribution of CCLWR grade values during (a) 2004, (b) 2007, (c) 2010, (d) 2013, (e) 2016 in 14 cities are shown in Figure 2.

In general, the CCLWR in Hunan Province presented a pattern of “four weeks high, central low” and showed a fluctuating growth trend ($\left|\bar{R_{n}}\right|=0.9998>W_{p}=0.703$). In 2004, the average value of CCLWR in the whole province was 0.3215. Zhangjiajie in the northwest of Hunan Province had the highest CCLWR value of 0.6280. In 2007, the CCLWR in the west increased significantly. The most significant decline in CCLWR was shown in Yueyang in the north of Hunan Province, decreasing from 0.2123 to 0.1517. In 2010, the CCLWR had a great improvement in the southeast region while it had a certain decline in the west. In 2013, the CCLWR of the whole province decreased in different degrees, among which the CCLWR of Changsha and Zhuzhou decreased from 0.3200 and 0.4248 in 2010 to 0.2770 and 0.3335 in 2013 respectively. In 2016, the CCLWR of the whole province was greatly improved with the average value of 0.4895. The CCLWR of Changsha increased from 0.2770 in 2013 to 0.4205 in 2016, with the most significant improvement. The highest value (0.8435) of CCLWR was in Zhangjiajie in the northwest, and the lowest value (0.2857) of CCLWR was in Xiangtan in the middle.

#### 3.2.2. Spatial and Temporal Distribution Characteristics of the Development Level of the Social Economy System

**Figure 3:**
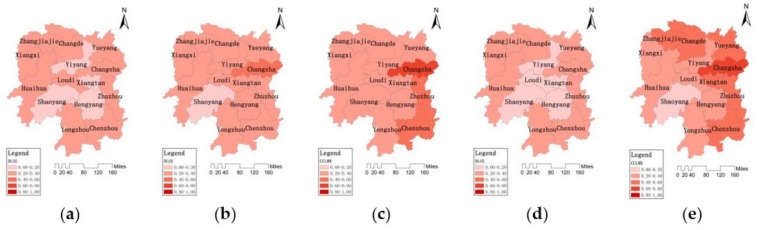
Temporal distribution of DLSE among 14 cities in Hunan Province during (**a**) 2004, (**b**) 2007, (**c**) 2010, (**d**) 2013, (**e**) 2016.

According to formula (8), the standardized indicator values and entropy weights in the social economy system can be used as the input data to calculate the development level of DLSE. DLSE takes several indicators into account, such as per capita income, urbanization rate, per capita GDP, and population density, to measure the comprehensive evaluation value of the development status of the regional social economy system. The larger the value of the comprehensive evaluation is, the higher the development level of the social economy system. The spatial and temporal distribution of DLSE grade values during (a) 2004, (b) 2007, (c) 2010, (d) 2013, (e) 2016 in 14 cities are shown in Figure 3.

In general, the DLSE in Hunan Province presented a pattern of “four weeks high, central low”, and showed a trend of continuous growth ($\left|\bar{R_{n}}\right|=0.9999>W_{p}=0.703$). In 2004, the DLSE of Hunan Province was at a low level, with the average DLSE of 0.1866. The DLSE of Loudi was the lowest with 0.1057. In 2007, the DLSE of the whole province increased slightly, and the average value of DLSE reached 0.2300. The DLSE of Loudi had the largest increase. In 2010, the DLSE of eastern and central cities presented a big increase. Only Shaoyang in the southwest remained at a low level, and its DLSE was only 0.1962. In 2013, the DLSE of the whole province continued to grow. The DLSE of Changsha, Zhuzhou, and Chenzhou in the east was in the leading position with 0.6327, 0.4453, and 0.4005, respectively. In 2016, the DLSE of the whole province was greatly improved and the average value reached 0.4142. The eastern regions with Changsha as the center had the fastest growth. From 2004 to 2016, the DLSE of Changsha, Zhuzhou, and Xiangtan increased from 0.1827 to 0.7646, from 0.2203 to 0.5175 and from 0.1201 to 0.4470 respectively with the fastest growth of DLSE in Hunan Province.

#### 3.2.3. Spatial and Temporal Distribution Characteristics of the Protection Level of the Ecological Environment System

**Figure 4:**
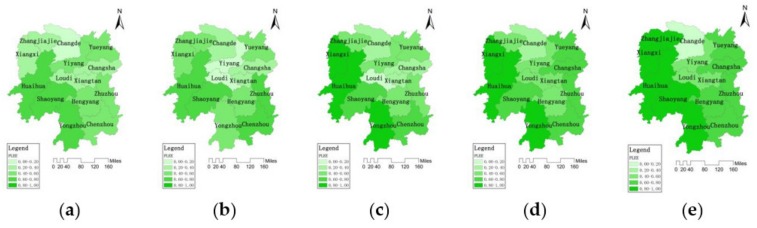
Spatial and temporal distribution of PLEE among 14 cities in Hunan Province during (**a**) 2004, (**b**) 2007, (**c**) 2010, (**d**) 2013, (**e**) 2016.

According to formula (9), the standardized indicator values and entropy weights in the ecological environment system can be used as the input data to calculate PLEE. PLEE takes indicators into account, such as wetland protection rate, COD concentration in the channel, forest coverage, ecological compliance rate of water functional zone to measure the comprehensive evaluation value of the protection status of the regional ecological environment system. The larger the value of the comprehensive evaluation is, the higher the protection level of the ecological environment system. The spatial and temporal distribution of PLEE grade values during (a) 2004, (b) 2007, (c) 2010, (d) 2013, (e) 2016 in 14 cities are shown in Figure 4.

In general, the PLEE in Hunan Province presented a pattern of “high in southwest, low in central and northeast” and showed a trend of continuous growth ($\left|\bar{R_{n}}\right|=0.9994>W_{p}=0.703$). In 2004, the average value of PLEE in Hunan Province was 0.4612. The PLEE of Yongzhou, Huaihua, and Shaoyang in the southwest reached above 0.70, while the PLEE of Changde in the north was only 0.1869. In 2007, the average PLEE of the whole province was 0.4827. The PLEE of Chenzhou and Zhuzhou in the southeast increased significantly, while the PLEE of Loudi and Yiyang in the middle decreased. The PLEE of Loudi was the lowest with 0.0972. In 2010, the PLEE of the whole province had a significant improvement. The PLEE of Huaihua and Xiangxi in the west reached 0.8970 and 0.8750, respectively. The PLEE of Loudi in the middle was still at a low level with 0.1247. In 2013, the PLEE of Hunan Province presented an overall improvement, with an average value of 0.6309. The PLEE of the southwest regions was above 0.50, while the PLEE of Changde in the north was lagging behind with only 0.3431. In 2016, the average PLEE of the whole province reached 0.7092. The PLEE of Changde and Yiyang was below 0.50, which was lagging behind other cities. The PLEE of Zhangjiajie and Xiangxi in the west reached 0.9560 and 0.9184, respectively, which was in the leading position in Hunan Province.

### 3.3. Spatial and Temporal Distribution Characteristics of the Coordinated Development Degree

**Figure 5:**
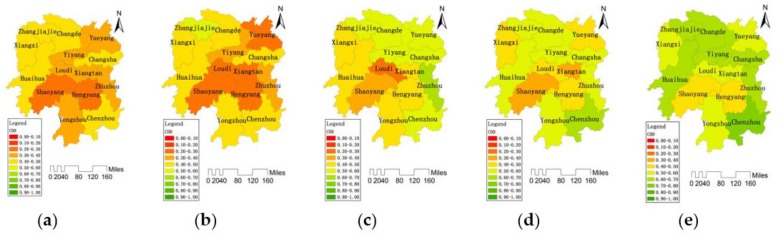
Spatial and temporal distribution of CDD among 14 cities in Hunan Province during (**a**) 2004, (**b**) 2007, (**c**) 2010, (**d**) 2013, (**e**) 2016.

According to the formula (10) and (11), the coordination degree (CD) and the development degree (DD) can be calculated respectively with the values of CCWR, DLSE, and PLEE as the input data. Combing with CD and DD, according to formula (12), the coordinated development degree (CDD) of the three systems can also be calculated. Coordination development degree (CDD) is a quantitative index that describes the development and coordination degree of various systems in a region under a certain spatial and temporal scale. The larger the value of the CDD is, the higher the coordinated development level of the three systems. The spatial and temporal distribution of CDD grade values during (a) 2004, (b) 2007, (c) 2010, (d) 2013, (e) 2016 in 14 cities are shown in Table 5 and Figure 5.

In general, the CDD of Hunan Province presented a pattern of “high on three sides, low in middle and southern areas”, and showed a trend of annual growth ($\left|\bar{R_{n}}\right|=0.9999>W_{p}=0.703$).

According to the classification and evaluation criteria of coordinated development shown in Table 2, we can divide the CDD of 14 cities in Hunan Province during (a) 2004, (b) 2007, (c) 2010, (d) 2013, (e) 2016 into different types of coordinated development as shown in Table 6.

According to Table 5, the average coordinated development degree of Hunan Province evolved from “Light disorder recession (V4)” in 2004 and 2007 to “Nearly disorder recession (V5)” in 2010, and then to “Reluctance coordinated development (V6)” in 2013 and 2016.

In 2004, Hengyang and Shaoyang in the southwest were in “Moderate disorder recession (V3)” Xiangtan, Loudi, Yiyang, and Yongzhou in the middle were in “Light disorder recession (V4)” Huaihua, Xiangxi, Zhangjiajie and Changdein the northwest and Changsha in the east were in “Nearly disorder recession (V5)”.

In 2007, Hengyang and Shaoyang in the southwest, Loudi in the middle and Xiangtan, Yueyang in the north were in “Moderate disorder recession (V3)” The western and southern regions of Hunan Province and Changsha in the east were in “Nearly disorder recession (V5)”.

In 2010, Loudi, in the middle, was still in “Moderate disorder recession (V3)”. The western and southern regions of Hunan Province were in “Nearly disorder recession (V5)” The northern regions of Hunan Province and Changsha in the east reached “Reluctance coordinated development (V6)” Zhuzhou in the east reached “Primary coordinated development (V7)”.

In 2013, Xiangtan and Shaoyang in the middle were in “Light disorder recession (V4)” Loudi and Hengyang in middle, Yueyang in the north and Xiangxi in the west were in “Nearly disorder recession (V5)” Chenzhou reached “Primary coordinated development (V7)” and other regions were in “Reluctance coordinated development (V6)”.

In 2016, Hengyang and Shaoyang in the middle were still in “Nearly disorder recession (V5)” Loudi and Xiangtan in the middle, Xiangxi in the west, Yueyang in the north and Yongzhou in the south were in “Primary coordinated development (V7)” Chenzhou in the southeast reached “Middle coordinated development (V8)”.

## 4. Discussion

According to above results, from 2004 to 2016, the average coordinated development degree of Hunan Province evolved from “Light disorder recession (V4)” to “Nearly disorder recession (V5)” then to “Reluctance coordinated development (V6)” Overall, the coordinated development in middle and southwest of Hunan Province was relatively backward, including Shaoyang, Hengyang, Loudi and Xiangtan. This was mainly due to the fact that the middle of Hunan Province was a hilly region surrounded by mountains in the south, east, and west, where the traffic was not convenient, which led to the backward social economy development in these areas. The coordinated development of Changsha, Zhuzhou, and Chenzhou in the east was in the leading position in the whole province. As Hunan provincial government took full advantage of its regional advantages and characteristics, a new pattern of regional development was formed. Changsha, Zhuzhou, and Xiangtan, as the leader of the “One Point and One Line” [23] area, gathered production factors and drove the enhancement of the Province′s economic development capacity.

In order to investigate the driving factor causing the distribution characteristic of the CDD, we need to analyze the coordinated development status of the water resources system, the social economic system, and the ecological environment system. Then, we need to diagnose which type of coordinated development state is in each region during various periods, and determine which subsequent state of the system is responsible for the regional discoordination.

**Figure 6:**
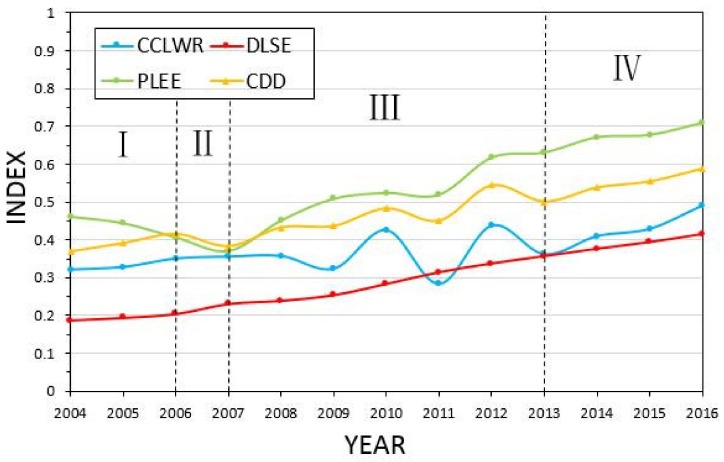
Trends of the average value of CCLWR, DLSE, PLEE, and CDD among 14 cities in Hunan Province from 2004 to 2016.

As shown in Figure 6, the CDD of the whole Province presented a rising trend with fluctuation. We can divide the development of Hunan Province from 2004 to 2016 into four stages.

1. Stage I (2004–2006): Social economy system restriction stage.

**Figure 7:**
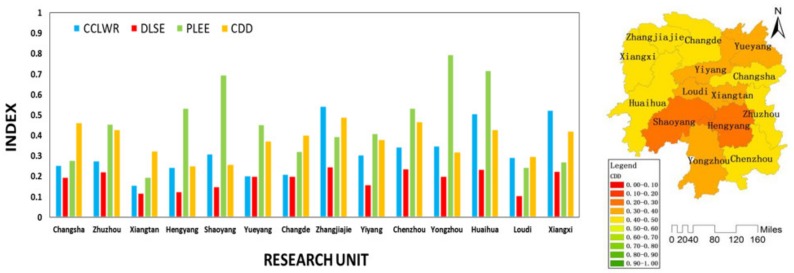
The CCLWR, DLSE, PLEE, and CDD of each unit in stage I.

As shown in Figure 6, during stage I, the CDD of the whole province was on the rise. During this period, the CCLWR basically remained stable, the DLSE showed an upward trend, while the PLEE showed a downward trend. This indicated the coordinated development of Hunan Province was mainly restricted by the backward social economy development. As shown in Figure 7, during stage I, the coordinated development in all cities was mainly restricted by the social economy system.

According to the “2006 Report on the Work of the Hunan Government” [49], this stage is one of the periods that Hunan Province′s social economy development was in the fast track. This proves that, in stage I, Hunan provincial government put the social economy development in a significant position in the provincial development planning to deal with the constraints of the social economy system.

It is worth noting that due to the rapid development of social economy, CDD of the whole province presented an increasing trend. In fact, Hunan Province achieved the development of the social economy system at the expense of the damage of the ecological environment. In previous studies, researchers often believed that the whole province was in a coordinated development state due to the rising trend of CDD from 2004 to 2006. However, according to this study, we found that the influence brought by the increase of CDD on the development of the social economy system was greater than that brought by the decrease of CDD on the degradation of the ecological environment system, which was not a sustainable way of development.

2. Stage II (2006–2007): Ecological environment system restriction stage.

**Figure 8:**
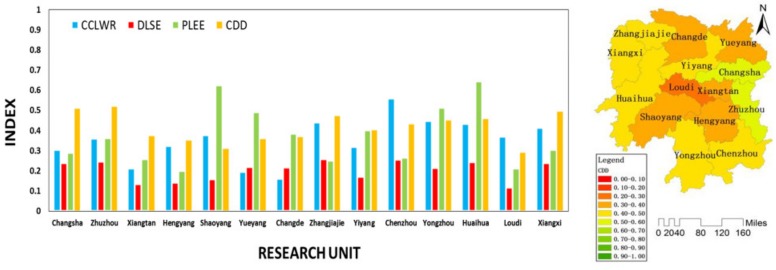
CCLWR, DLSE, PLEE, and CDD of each unit in the stage II.

As shown in Figure 6, during stage II, the CDD of the whole province showed a declining trend. During this period, the CCLWR remained basically stable, the DLSE showed an upward trend, and the PLEE showed a downward trend. This indicated that although the social economy system and the water resources system were in a stable development process, the coordinated development of Hunan Province presented a downward trend. When the ecological environment system continued to degrade from the previous stage to a certain extent, it began to become the main factor restricting the coordinated development of regions. As shown in Figure 8, during the stage II, the coordinated developments in Changsha, Zhuzhou, Xiangtan, Hengyang, Shaoyang, Yiyang, Chenzhou, Yongzhou, Huaihua Loudi, and Xiangxi were mainly restricted by the social economy system. The coordinated developments in Yueyang and Changde were mainly restricted by the water resources system. The coordinated development in Zhangjiajie was mainly restricted by the ecological environment system.

According to “2007 Report on the Work of the Hunan Government” [50], in this stage, Hunan provincial government strengthened the ecological construction and environmental protection. Hunan provincial government focused on controlling the industrial and mining pollution in Dongting Lake and Xiangjiang River. Meanwhile, decision-maker carried out major ecological projects and environmental improvement projects, and strengthened protection and management of forests, wetlands, and water resources. This proved that in stage II, Hunan provincial government put the ecological environment protection in a significant position in the provincial development planning to deal with the constraints of the ecological environment system.

3. Stage III (2006–2007): Water resources system restriction stage.

**Figure 9:**
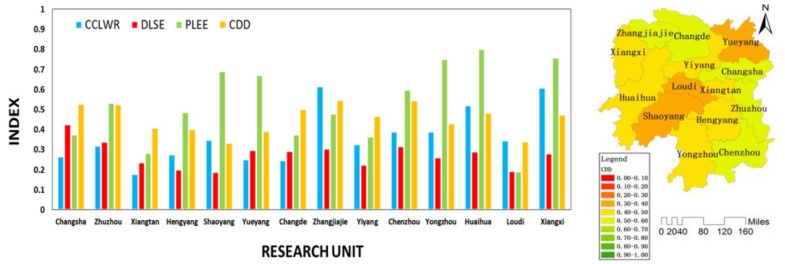
CCLWR, DLSE, PLEE, and CDD of each unit in stage III.

As shown in Figure 6, during stage III, the DLSE and the PLEE in Hunan Province showed a steady growth trend. The CCLWR in Hunan Province fluctuated greatly with the inter-annual change, while the CDD was mainly restricted by the water resource system and fluctuated with the CCLWR. This phenomenon was mainly due to the large inter-annual variations of rainfall and available water resources in Hunan Province, and the uneven temporal and special distribution of water resources. As shown in Figure 9, during stage III, the coordinated developments in Changsha, Zhuzhou, Xiangtan, Yueyang, and Changde were mainly restricted by water resources system. The coordinated developments in Hengyang, Shaoyang, Yiyang, Chenzhou, Yongzhou, Huaihua, Zhangjiajie, and Xiangxi were mainly restricted by social economy system. The coordinated development in Loudi was mainly restricted by ecological environment system.

According to the “2013 Report on the Work of the Hunan Water Resources Department” [51], rainfall in Hunan Province from 2007 to 2013 was not evenly distributed in time and space, and flood and drought disasters occurred frequently. Hunan provincial government accelerated the construction of major water conservancy projects and added a closed protective ring for flood control in cities. Decision-maker improved the systems and mechanisms for preventing, mitigating, and providing disaster relief, fully implemented the responsibility system for flood control and drought relief, and revised and improved flood control plans for hidden dangers of reservoirs and mountain floods. This proved that in stage III, Hunan provincial government put water resources management in a significant position in the provincial development planning to deal with the constraints of the water resources system.

4. Stage IV (2013–2016): Reluctance coordinated development stage.

**Figure 10:**
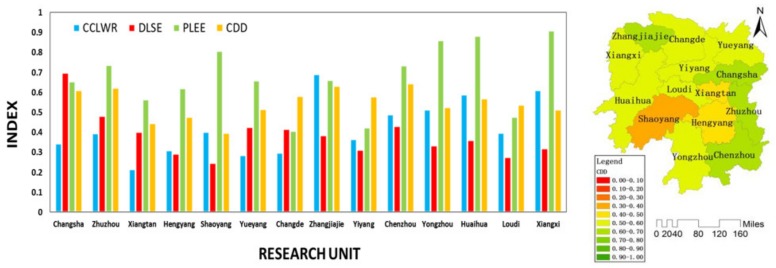
The CCLWR, DLSE, PLEE, and CDD of each unit in stage IV.

As shown in Figure 6, during stage IV, the CCLWR, DLSE, and PLEE of the whole province showed an upward trend and the CDD of whole province also presented a stable growth trend. As shown in Figure 10, during stage IV, the coordinated developments in Changsha, Zhuzhou, Xiangtan, Yueyang, and Changde were mainly restricted by water resources system. The coordinated developments in Hengyang, Shaoyang, Zhangjiajie, Yiyang, Chenzhou, Yongzhou, Huaihua, Loudi, and Xiangxi were mainly restricted by the social economy system.

According to the “2016 Report on the Work of the Hunan Government” [52], the economic scale of Hunan Province continued to expand at this stage, entered into stability, and made progress, and its development level continuously improved. Hunan provincial government implemented the strictest mechanism for managing water resources and conserving land, and strictly adhered to the upper limit of resource consumption, the bottom line of environmental quality, ecological protection, and the red line of arable land. This proved that the Hunan provincial government was committed to the sustainable coordinated development among the social economy system, the water resource system, and the ecological environment system, and Hunan Province was gradually transformed into a reluctance coordinated development stage.

## 5. Conclusions

In this paper, we first collected and analyzed data from 14 municipal areas from 2004 to 2016 in Hunan Province. Then we established the evaluation system of CCLWR, DLSE, and ecological PLEE, respectively. Based on the comprehensive development evaluation among three systems, we calculated the coordinated development degree of all research units using the coordinated development theory. Then, we analyzed the spatial and temporal distribution characteristics of the CCLWR, DLSE, PLEE, and CDD in Hunan Province, and draws the conclusion as follows:

1. The CCLWR in Hunan Province from 2004 to 2016 presented a pattern of “high in the west and low in the east” and showed a fluctuating growth trend. The fluctuation of CCLWR was mainly affected by the changes in total water resources per unit, water consumption per 10^4^ CNY of GDP and annual rainfall. Zhangjiajie, Xiangxi and Huaihua in the west region were rich in water resources, while Changsha and Zhuzhou in the east had fewer water resources. Therefore, decision-makers need to put forward a scientific management program to solve the uneven temporal and spatial distribution of water resources and ensure healthy and sustainable development of the water resources system.
2. The DLSE in Hunan Province from 2004 to 2016 presented a pattern of “high on all sides, low in the middle” and showed a continuous growth trend. The growth of DLSE was mainly affected by the changes of urbanization rate, population density, and per capita GDP. As the capital of Hunan Province, Changsha was the most vigorous growth point and gathering place of regional economic development. The rapid growth of the social economy system in Changsha radiated the development of surrounding city clusters, such as Zhuzhou and Xiangtan. However, due to the unscientific urban management in the process of economic development, the ecological environment in some cities was damaged to some extent. Therefore, decision-makers need to adjust the industrial layout and find a sustainable development model.
3. The PLEE in Hunan Province from 2004 to 2016 presented a pattern of “high in southwest, low in central and low in northeast” and showed a fluctuating growth trend. The PLEE growth was mainly affected by the change of ecological compliance rate of water functional zone and wetland protection rate. From 2004 to 2007, the PLEE of the whole province continued to decline, mainly due to the one-sided pursuit of social economy development and the neglect of the importance of the ecological environment. After 2007, decision-makers began to pay attention to the protection of the ecological environment, and the province′s ecological environment quality was greatly improved. The ecological environment protection in Xiangxi, Zhangjiajie, Huaihua achieved remarkable results, while the level of ecological environment protection in Changsha, Changde, and other areas still needs to be improved.
4. The CDD of Hunan Province from 2004 to 2016 evolved from “Light disorder recession (V4)” to “Nearly disorder recession (V5)”, then to “Reluctance coordinated development (V6)”, which presented a fluctuating growth trend. The CDD was restricted by different systems in different development periods and had a large regional difference. From 2004 to 2006, Hunan Province was in the social economy system restriction stage. Due to the rapid development of social economy, the CDD of the whole province presented a growth trend during this period. However, the ecological environment was destroyed at the same time. From 2006 to 2007, Hunan Province was in the ecological environment system restriction stage. Due to the destruction, the ecological environment system was degraded to a certain extent and began to restrict the coordinated development of the whole province. From 2007 to 2013, Hunan Province was in the water resources system restriction stage. During this period, the rainfall and available water resources in Hunan Province vary greatly from year to year, and the distribution of water resources was not uniform in time and space. As a result, the coordinated development of the whole province was mainly restricted by the water resources system. From 2013 to 2016, Hunan Province was in the reluctance coordinated the development stage. With the rapid development of social economy, the protection of the ecological environment and the management of water resources were also improved. Therefore, Hunan Province began to be in a stable and sustainable coordinated development process.

In summary, in order to promote the overall coordinated development of the region, decision-makers should adjust measures to local conditions for the determination of the coordinated development mode. In recent years, the coordinated development of Changsha, Zhuzhou, Xiangtan, Yueyang, and Changde lagged in water resources system. Therefore, the decision-makers should carry out sustainable water resources management in these cities. Decision-makers can develop efficient water-saving industries by adjusting the industrial structure and the distribution of productivity and controlling the growth of water resource demands. The coordinated development of Shaoyang, Yongzhou, Huaihua, Loudi, and Xiangxi was mainly restricted by the social economy system. Since these areas are located in mountainous and hilly areas with beautiful natural scenery, decision-makers can take full advantage of the local ecological environment in these cities. The vigorous development of the tourism industry [53] can significantly stimulate the regional social economy development in these areas.

## Figures and Tables

**Table 1:** The comprehensive development evaluation index system of the three systems.

System Layer	Indicator Layer	Properties	Data Source
Water resources system	Per capita water consumption (10^4^m³/person) $\omega_{WR,1}$	+	[26]
	Water consumption per 10^4^ CNY of GDP (m³/10^4^ CNY) $\omega_{WR,2}$	−	[26]
	Annual rainfall (mm) $\omega_{WR,3}$	+	[26]
	Per capita water resources (10^4^m³/person) $\omega_{WR,4}$	−	[26]
	Total water resources per unit (10^9^ m³) $\omega_{WR,5}$	+	[26]
Social economy system	Per capita income (CNY) $\omega_{SE,1}$	+	[27]
	Urbanization rate (%) $\omega_{SE,2}$	+	[27]
	Per capita GDP (CNY) $\omega_{SE,3}$	+	[27]
	Population density (person/ km^2^) $\omega_{SE,4}$	−	[27]
Ecological environment system	Wetland protection rate (%) $\omega_{EE,1}$	+	[28]
	COD concentration in the channel (mg/L) $\omega_{EE,2}$	−	[26]
	Forest coverage (%) $\omega_{EE,3}$	+	[28]
	Ecological compliance rate of water functional zone (%) $\omega_{EE,4}$	+	[26]

**Table 2:** Classification and evaluation criteria of coordinated development.

Category	CDD	Subclass
Coordination category	0.90~1.00	High coordinated development (V10)
	0.80~0.89	Good coordinated development (V9)
	0.70~0.79	Middle coordinated development (V8)
Transition category	0.60~0.69	Primary coordinated development (V7)
	0.50~0.59	Reluctance coordinated development (V6)
	0.40~0.49	Nearly disorder recession (V5)
Imbalanced recessional category	0.30~0.39	Light disorder recession (V4)
	0.20~0.29	Moderate disorder recession (V3)
	0.10~0.19	Serious disorder recession (V2)
	0.00~0.09	Extreme disorder recession (V1)

**Table 3:** Weight calculation results of the comprehensive development evaluation of the three systems.

System Layer	Indicator Layer	Properties	Weight
Water resources system	Per capita water consumption (10^4^ m³/person) $\omega_{WR,1}$	+	0.1039
	Water consumption per 10^4^ CNY of GDP (m³/10^4^ CNY) $\omega_{WR,2}$	−	0.3145
	Annual rainfall (mm) $\omega_{WR,3}$	+	0.1484
	Per capita water resources (10^4^ m³/person) $\omega_{WR,4}$	−	0.0684
	Total water resources per unit (10^9^ m³) $\omega_{WR,5}$	+	0.3649
Social economy system	Per capita income (CNY) $\omega_{SE,1}$	+	0.1531
	Urbanization rate (%) $\omega_{SE,2}$	+	0.4514
	Per capita GDP (CNY) $\omega_{SE,3}$	+	0.0460
	Population density (person/km^2^) $\omega_{SE,4}$	−	0.3747
Ecological environment system	Wetland protection rate (%) $\omega_{EE,1}$	+	0.3191
	COD concentration in the channel (mg/L) $\omega_{EE,2}$	−	0.2047
	Forest coverage (%) $\omega_{EE,3}$	+	0.1047
	Ecological compliance rate of water functional zone (%) $\omega_{EE,4}$	+	0.3715

**Table 4:** The critical value of Spearman’s rank correlation coefficient.

*n*	Significance Level			
	0.10	0.05	0.02	0.01
12	0.503	0.587	0.671	0.727
13	0.484	0.560	0.648	0.703
14	0.464	0.538	0.622	0.675

**Table 5:** The value of the CDD among 14 cities in Hunan Province during (**a**) 2004, (**b**) 2007, (**c**) 2010, (**d**) 2013, (**e**) 2016.

CITIES	(a) 2004	(b) 2007	(c) 2010	(d) 2013	(e) 2016	Rn
Changsha	0.4386	0.4703	0.5619	0.5262	0.6903	0.9999
Zhuzhou	0.3519	0.3138	0.6183	0.5757	0.6805	0.9997
Xiangtan	0.3035	0.2635	0.4803	0.3433	0.5451	0.9999
Hengyang	0.2408	0.2915	0.4095	0.4612	0.4993	0.9999
Shaoyang	0.2292	0.2916	0.3679	0.3609	0.4280	1.0000
Yueyang	0.3983	0.2760	0.5231	0.4310	0.5611	0.9998
Changde	0.4463	0.4335	0.5524	0.5604	0.6263	0.9999
Zhangjiajie	0.4619	0.5088	0.5177	0.5967	0.6327	0.9999
Yiyang	0.3704	0.3819	0.5042	0.5417	0.6087	0.9999
Chenzhou	0.4474	0.4808	0.5614	0.6208	0.7056	0.9999
Yongzhou	0.3272	0.4379	0.4460	0.5046	0.5546	0.9998
Huaihua	0.4392	0.4913	0.4683	0.5206	0.6036	0.9999
Loudi	0.3043	0.2570	0.2866	0.4906	0.5681	0.9998
Xiangxi	0.4140	0.4712	0.4733	0.4891	0.5401	1.0000
AVG	0.3695	0.3835	0.4836	0.5016	0.5889	0.9999

**Table 6:** The evaluation types of the CDD among 14 cities in Hunan Province during (**a**) 2004, (**b**) 2007, (**c**) 2010, (**d**) 2013, (**e**) 2016.

CITIES	(a) 2004	(b) 2007	(c) 2010	(d) 2013	(e) 2016
Changsha	V5	V5	V6	V6	V7
Zhuzhou	V4	V4	V7	V6	V7
Xiangtan	V4	V3	V5	V4	V6
Hengyang	V3	V3	V5	V5	V5
Shaoyang	V3	V3	V4	V4	V5
Yueyang	V4	V3	V6	V5	V6
Changde	V5	V5	V6	V6	V7
Zhangjiajie	V5	V5	V6	V6	V7
Yiyang	V4	V4	V6	V6	V7
Chenzhou	V5	V5	V6	V7	V8
Yongzhou	V4	V5	V5	V6	V6
Huaihua	V5	V5	V5	V6	V7
Loudi	V4	V3	V3	V5	V6
Xiangxi	V5	V5	V5	V5	V6
AVG	V4	V4	V5	V6	V6
